# Cholelithiasis and cholecystitis in children and adolescents: Does this increasing diagnosis require a common guideline for pediatricians and pediatric surgeons?

**DOI:** 10.1186/s12876-021-01772-y

**Published:** 2021-04-21

**Authors:** Sonja Diez, Hanna Müller, Christel Weiss, Vera Schellerer, Manuel Besendörfer

**Affiliations:** 1 Friedrich-Alexander-Universität Erlangen-Nürnberg (FAU), Pediatric Surgery, Department of Surgery, University Hospital Erlangen, Erlangen, Germany; 2 Friedrich-Alexander-Universität Erlangen-Nürnberg (FAU), Neonatology and Intensive Care Unit, Children’s Hospital Erlangen, University Hospital Erlangen, Erlangen, Germany; 3 Philipps-Universität Marburg, Neonatology and Pediatric Intensive Care, Department of Pediatrics, University of Marburg, Marburg, Germany; 4 Ruprecht-Karls-Universität Heidelberg, Department of Medical Statistics and Biomathematics, Medical Faculty Mannheim, Heidelberg University, Mannheim, Germany; 5 Friedrich-Alexander-Universität Erlangen-Nürnberg (FAU), General and Visceral Surgery, Department of Surgery, University Hospital Erlangen, Erlangen, Germany

**Keywords:** Pediatric gallstones, Cholecystitis, Symptomatic cholecystolithiasis

## Abstract

**Background:**

In contrast to adults, for whom guidelines on the cholelithiasis treatment exist, there is no consistent treatment of pediatric patients with cholelithiasis throughout national and international departments, most probably due to the lack of evidence-based studies.

**Methods:**

We evaluated the German management of pediatric cholelithiasis in a dual approach. Firstly, a retrospective, inter-divisional study was established, comparing diagnostics and therapy of patients of the pediatric surgery department with the management of patients aged < 25 years of the visceral surgery department in our institution over the past ten years. Secondarily, a nation-wide online survey was implemented through the German Society of Pediatric Surgery.

**Results:**

Management of pediatric patients with cholelithiasis was primarily performed by pediatricians in the retrospective analysis (*p* < 0.001). Pediatric complicated cholelithiasis was not managed acutely in the majority of cases with a median time between diagnosis and surgery of 22 days (range 4 days–8 months vs. 3 days in visceral surgery subgroup (range 0 days–10 months), *p* = 0.003). However, the outcome remained comparable. The hospital’s own results triggered a nation-wide survey with a response rate of 38%. Primary pediatric medical management of patients was confirmed by 36 respondents (71%). In case of acute cholecystitis, 22% of participants perform a cholecystectomy within 24 h after diagnosis. Open questions revealed that complicated cholelithiasis is managed individually.

**Conclusions:**

The management of pediatric cholelithiasis differs between various hospitals and between pediatricians and pediatric surgeons. Evidence-based large-scale population studies as well as a common guideline may represent very important tools for treating this increasing diagnosis.

**Supplementary information:**

The online version contains supplementary material available at 10.1186/s12876-021-01772-y.

## Background

The incidence of cholelithiasis in children and adolescents appears to be increasing, even if the entity remains to be a rare disease within this population [1]. Prevalence is ranking between 0.13 and 1.9% [2]. Diagnostic and therapeutic management seems to be heterogeneous in clinical practice and appears to be based on small population studies [3, 4]. In contrast, different guidelines apply to adults in various countries (e.g., Europe [5], Japan [6], USA [7]). In Germany, guidelines by the German Societies for Digestive and Metabolic Diseases and for Surgery of the Alimentary Tract regulate the management of cholelithiasis in adults [8]. Separate treatment guidelines in the pediatric sector on the basis of evidence-based large-scale population studies are either lacking, outdated or represent expert opinions of distinct hospitals (e.g., Sweden [2], Brazil [9], India [10], USA [11], Egypt [12], Iran [13]). This is significant with regard to the timing of surgery. The guidelines for adult management updated in 2018 are based on the largest randomized trial in adults so far, the multi-center “Acute cholecystitis: early versus delayed cholecystectomy” trial [8]. Even though the results are being controversially discussed [14], they recommend an early cholecystectomy within the first 48 h of symptoms of acute cholecystitis and an elective cholecystectomy in symptomatic cases without signs of inflammation.

Aiming at assessing the management and especially the surgical timing of this rare but increasing pediatric disease, we chose a dual approach in this study. Firstly, a retrospective analysis of patients with cholecystectomy was performed within our institution, comparing diagnostics and therapy of children and adolescents in the pediatric surgery department with the management of young patients (aged < 25 years) in the visceral surgery department. Secondarily, diagnostic and therapeutic standards of this entity were evaluated in pediatric patients using an online survey via the German Society of Pediatric Surgery.

## Patients and methods

### Structure of the retrospective study

#### Data collection and inclusion criteria

A review of all patients aged between 0 and 25 years was conducted, who underwent cholecystectomy at our institution during the period of January 2009 to December 2019. Data for this retrospective, inter-divisional study was obtained by reviewing the medical and imaging records of medical histories of all patients. Patients were identified by searching for all surgical reports including cholecystectomy within this time period. We defined inclusion criteria for all patients who underwent cholecystectomy due to cholelithiasis and/or cholecystitis with classical signs of inflammation (see below) up to the age of 25 years. All cases of cholecystectomies of the pediatric surgery department and all cases of cholecystectomies of the visceral surgery were analyzed. Accordingly, cases with gallbladder polyps or cases in which cholecystectomy was performed along with other procedures were excluded. For investigation of diagnostic and therapeutic differences, patients were divided into subgroups of cases of the pediatric surgery and cases of the visceral surgery department.

#### Patients’ clinical characteristics, diagnostics and treatment

Demographic baseline data were recorded, including predisposing factors for cholelithiasis and the clinical presentation of patients. Patients’ weight was hereby evaluated to estimate the influence of obesity on cholelithiasis with regard to their height. Patients were classified based on the body-mass-index (BMI) according to the WHO definition [15]. In children and adolescents (< 18 years of age), BMI was assessed according to percentiles: overweight was present > 90th percentile, obesity > 97th percentile. Classification into percentiles had to be particularly applied to three patients of the visceral surgery group, being < 18 years of age at time of surgery. In patients ≥ 18 years of age, overweight was solely defined as BMI > 25 kg/m^2^, and obesity as BMI > 30 kg/m^2^. Indications for observational treatment with or without antibiotics and analgesia, for treatment with ursodesoxycholic acid (UDCA) to induce litholysis, and for cholecystectomy were explored. For patients treated with UDCA, therapy was considered effective in cases of complete dissolution of gallstones and disappearance of clinical symptoms.

#### Classification of asymptomatic cholelithiasis, uncomplicated (symptomatic) and complicated cholelithiasis

Only patients with incidental findings of cholelithiasis without any symptoms at the time of diagnosis were classified as asymptomatic patients. For further analysis of timing of surgery, patients were grouped as follows: Patients with symptomatic cholelithiasis, including diffuse abdominal pain and colics, nausea and vomiting without signs of inflammation or other complications were categorized as uncomplicated symptomatic cholelithiasis. Complicated cholelithiasis was defined in cases of choledocholithiasis or biliary pancreatitis. We additionally included cases of acute cholecystitis (acute pain in the right upper quadrant, accompanied by systemic inflammatory signs such as fever, increased white blood cell count, increased C-reactive protein (CrP)) in the group of complicated cholelithiasis.

#### Ethical approval

Both parts of the study were approved by the local ethics committee (Ethikkommission der Friedrich-Alexander-Universität Erlangen-Nürnberg) in accordance with the declaration of Helsinki (1964) and its later amendments (reference number 164_20 Bc). The local ethics committee did not demand informed consent due to retrospective analysis of anonymized data.

### Online survey

**Fig. 1:**
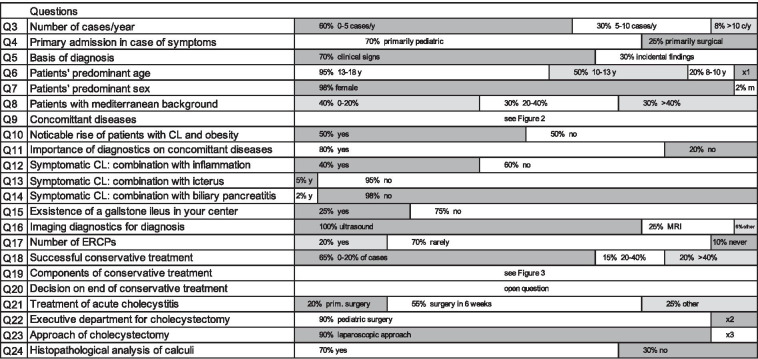
Questionnaire of the online survey, including given answers. Question 1/2 contained demographical information about the participants. Values have been rounded for clarity. Annotations: × 1: “10% 6–8 y”; × 2: “2% visceral surgery, 8% combined”; × 3: “10% mainly laparoscopic approach”. The full online survey is presented as part of the Additional file 1 of this manuscript. *CL* cholelithiasis, *ERCP* endoscopic retrograde cholangiopancreatography

Based on the results of the retrospective data of our monocentric study, a web-based questionnaire was designed and implemented using an online platform for surveys (SurveyMonkey Inc., San Mateo, California, United States). Data was collected and classified with regard to structural, epidemiological, diagnostic and therapeutic management of pediatric cholelithiasis. The online questionnaire consisted of 24 items. Except for 2 questions, all questions were closed (8 dichotomous, 14 multiple choice, 5 of which with multiple possible answers). Six closed questions included the possibility of adding textual remarks. The questionnaire is presented in Fig. 1 and can be found in whole in the Additional file 1.

Pediatric surgery departments were identified and contacted via the German Society for Pediatric Surgery (DGKC). We invited all 133 pediatric surgery departments at hospitals as well as pediatric surgeons with reserved beds at hospitals to participate in our survey in January 2020 and reminded potential participants to complete the survey at the beginning of March 2020. Responses received between January and March 15^th^, 2020 were eligible for inclusion. No financial compensation was granted and secure sockets layer (SSL)-secured data transmission was ensured.

### Data quality and statistical analyses

Statistical analyses of patients within the retrospective single-center study were conducted using SAS software release 9.4 (SAS Institute Inc., Cary, NC, USA). Quantitative variables are presented by median and range. For qualitative factors, absolute and relative frequencies are given. Non-parametric Mann–Whitney-*U* tests have been performed in order to compare two groups regarding a quantitative variable. For categorical factors, *χ*^2^ test has been applied. However, if the conditions of the Chi^2^-test were not fulfilled (i.e., one of the under the null hypothesis expected frequencies was less than 5), Fisher’s exact test has been used instead. A *p* value < 0.05 was considered to be statistically significant.

Within the online survey, all completed responses were included in the analysis. Answers were exported from the online platform as raw and summarized data. Results are presented as absolute frequency (n of x respondents) and proportion (%).

## Results

### Results of the retrospective analysis, comparing management of pediatric and visceral surgery

A total of 87 patients with cholelithiasis was treated at our university center. Demographic baseline data are presented in Table 1. Weight and height of the pediatric patients were assessed, resulting in a median percentile of BMI of 78.0 (range 1–100) with 2 overweighted patients (> 90th percentile) and further 7 obese patients (> 97th percentile). As described above, obesity in children and adolescents was classified according to percentiles and in adult patients solely according to BMI (26.3 ± 7 kg/m^2^, range 15–48.5 kg/m^2^), whereby overweight and obesity could be seen significantly more frequently in the visceral surgery subgroup of patients (*p* < 0.001). While only a trend of higher pediatric percentage concerning concomitant disorders could be confirmed in the retrospective analysis (*p* = 0.163), significant associations could be observed for single concomitant diseases (*p* = 0.003 for hemolytic disorders and *p* = 0.021 for total/partial parenteral nutrition).

**Table 1:** Baseline demographic data

	Pediatric surgery group (n = 34)	Visceral surgery group (n = 53)	*p* value	Test
Age at surgery [in years: median (range)]	15 (7–17)	23 (15–25)		
Sex [n (%)]			0.687	Chi-square
Male	11 (32%)	15 (28%)		
Female	23 (68%)	38 (72%)		
Index admission [n (%)]			< 0.001*	Chi-square
Internal/pediatric medical	25 (74%)	12 (23%)		
Surgical/pediatric surgical	9 (26%)	41 (77%)		
Concomitant diagnoses [n (%)]			0.163	Chi-square/Fisher
No other diagnosis	16 (47%)	33 (62%)		
Concomitant diagnoses	18 (53%)	20 (38%)		
Spherocytosis/hemolytic disorder	7 (20%)	0	0.001	
Total/partial parenteral nutrition	4 (12%)	0	0.021	
Reduction of weight	2 (6%)	3 (6%)	1.000	
Malignancy	1 (3%)	2 (4%)	1.000	
Other (inter alia cerebral palsy, renal transplantation, myasthenia gravis, musc. dystrophies, Behcet’s disease)	4 (12%)	15 (28%)	0.090	
Weight at surgery [n (%)]			< 0.001*	Fisher
Normal weight	28 (82%)	22 (42%)		
Overweight	0	18 (34%)		
Obesity	6 (18%)	13 (24%)		

Summary of baseline data comparing patients of the pediatric surgery group (children and adolescents) with patients of the visceral surgery group (adolescents and young adults aged ≤ 25 years)

Significant values are indicated by an asterisk

Primary admission of patients differed significantly in comparison of subgroups: 25 out of 34 pediatric patients (74%) were diagnosed and treated primarily pediatric-medically and were presented to the pediatric surgery in the course of time, irrespective of status of symptoms or complications. In contrast, 77% of adult patients (41/53 cases) were treated primarily by the visceral surgery department (*p* < 0.001). Surgical timing and therapeutic approach of the whole study population are depicted in Table 2. Here we aimed at describing an overview of patients’ management in contrast of the study’s subgroups, as no further differentiation of cases was made at this point of the analysis. No differences on surgical timing or in the course of time (e.g., after the publication of the ACDC study) were seen in cases of symptomatic cholelithiasis. Conservative, observatory treatment (with or without a combination of analgesia, proton pump inhibitors and antibiotics at first signs of cholecystitis) was started in 33/34 pediatric patients (97%) and in 35/53 visceral surgical cases (66%). In 7 pediatric patients (21%), litholysis was induced by treatment with UDCA, which was not considered effective in any patient of the cohort. Complications before surgery occurred more frequently in the pediatric surgery subgroup: a biliary pancreatitis was seen in 8 pediatric patients (24%, vs. 3 cases of the visceral surgery subgroup (6%), *p* = 0.021). Choledocholithiasis was confirmed in 6 cases of the pediatric surgery subgroup (18%) and in 5 patients of the visceral surgery subgroup (9%, *p* = 0.318). Colics and cholestasis were analyzed apart from further complications and were seen in almost every patient of the two subgroups (31/34 pediatric patients (91%) and 48/53 visceral surgery patients (91%), *p* = 1.000). Occurrence of further complications is presented in Table 2. Complicated cases were not increased in pediatric patients (*p* = 0.390), although biliary pancreatitis was seen significantly more often in pediatric CL patients (*p* = 0.021). Postsurgical complications, such as infections or digestive disorders, were not seen in the pediatric subgroup and in only two patients of the visceral surgery subgroup (involving one patient with bilious drainage and another with a postsurgical abscess).

**Table 2:** Therapeutic management

	Pediatric surgery group (n = 34)	Visceral surgery group (n = 53)	*p* value	Test
Time of symptoms [in months: median (range)]	4 (0–41)	1 (0–69)	0.075	*U* test
Time between diagnosis and surgery [median (range)]	15 days (0–12 months)	4 days (0–12 months)	0.128	*U* test
Complicated cases [n (%)]			0.390	Chi-square
Yes	11 (32%)	22 (42%)		
No	23 (68%)	31 (58%)		
Complications [n (%), multiple answers possible]				
Acute cholecystitis	8 (24%)	20 (38%)	0.116	Chi-square
Cholangitis/choledocholithiasis	6 (18%)	5 (9%)	0.327	Fisher
Pancreatitis	8 (24%)	3 (6%)	0.021*	Fisher
Approach [n (%)]			1.000	Fisher
Laparoscopic	30 (88%)	46 (87%)		
Open	2 (6%)	4 (8%)		
Conversion	2 (6%)	3 (6%)		
Duration of surgery [in minutes: median (range)]	136 (45–337)	86 (33–198)	< 0.001*	*U* test
Diagnosis in histopathological evaluation [n (%)]			0.093	Fisher
Acute inflammation	1 (3%)	10 (19%)		
Chronic inflammation	27 (79%)	35 (66%)		
Acute and chronic inflammation	6 (18%)	8 (15%)		

Therapeutic management of symptomatic cholecystolithiasis (complicated and uncomplicated cases) in the group of pediatric surgery patients in comparison with the group of visceral surgery patients

Significant results are indicated by an asterisk

Assessment of surgical management and timing was performed according to classification of complicated and uncomplicated cholelithiasis stated above. Differences in clinical management of complicated cases are presented in Table 3.

**Table 3:** Management of complicated cholelithiasis

	Pediatric surgery, complicated cases (n = 11, 32% of pediatric surgery group)	Visceral surgery, complicated cases (n = 22, 42% of visceral surgery group)	*p* value	Test
Age at surgery [in years: median (range)]	14 (7–17)	23 (18–25)		
Index admission [n (%)]			< 0.001*	Fisher
Internal/pediatric medical	8 (73%)	7 (32%)		
Surgical/pediatric surgical	3 (27%)	15 (68%)		
Time of symptoms [median (range)]	58 days (4 days–41 months)	11 days (1 day–31 months)	0.119	*U* test
Complications [n (%), multiple answers possible]				
Acute cholecystitis	8 (73%)	20 (91%)	0.304	Fisher
Choledocholithiasis	6 (55%)	5 (23%)	0.117	Fisher
Biliary pancreatitis	8 (73%)	3 (14%)	0.001*	Fisher
Time between diagnosis and surgery [median (range)]	22 days (4 days–8 months)	3 days (0 days–10 months)	0.003*	*U* test
Timing of surgery after diagnosis [n (%)]			< 0.001*	Fisher
Surgery within day 0–4	1 (9%)	17 (77%)		
Surgery within day 5–42	6 (55%)	1 (5%)		
Surgery day > 42	4 (36%)	4 (18%)		
C-reactive protein (CrP) at surgery [in mg/l: median (range)]	1 (0–167)	30 (0–221)	0.004*	*U* test
Number of hospital days after surgery [in days: median (range)]	3 (3–8)	3 (2–11)	0.401	*U* test

Management of complicated cholelithiasis in comparison of patients of the pediatric surgery group (children and adolescents) with patients of the visceral surgery group (adolescents and young adults aged ≤ 25 years)

Significant values are indicated by an asterisk

### Results of the online survey

We received 51 completed responses, resulting in a response rate of 38%. 23% of the respondents are working at a university hospital (n = 12), 67% at a non-university hospital (n = 34), and 10% at medical care centers with reserved hospital beds (n = 5).

**Fig. 2:**
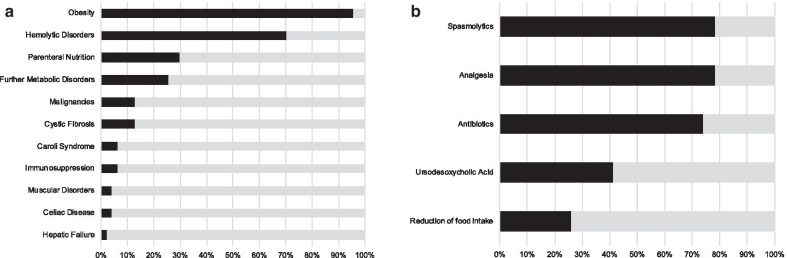
**a** Answers to question Q9: Results of the online survey due to concomitant disorders of cholelithiasis (answered by 47 respondents (92%)). **b** Answers to question Q19: Composition of conservative treatment of cholecystitis with cholelithiasis according to the results of the online survey (answered by 46 respondents (90%))

The questionnaire is presented in Fig. 1 and comprised demographic (Q3–Q11), diagnostic (Q5–Q17) and therapeutic data (Q18–Q24) (see also Additional file 1 for the questionnaire in whole). The majority of the surveyed institutions treat 0–5 patients/year (n = 31, 61%, see Q3). Diagnosis of cholelithiasis is rare in younger children and increases with progressing age (see Q6). An increase of diagnosis in obese children and adolescents over the past years was not noticeable in clinical practice in 49% of the institutions (n = 25, see Q10). Distribution of concomitant diagnoses of the online survey is presented in Fig. 2a (Q9). Primary pediatric medical management of cholelithiasis could be confirmed by 36 respondents (71%, see Q4). Routine diagnostics in cases with epigastric pain included sonography in 100% (n = 47/47) and MRI in 23% (n = 11/47) according to the answer of survey’s Q16, whereas CT was not included in routine diagnostics. Figure 2b illustrates the most common options of conservative treatment of pediatric patients with cholelithiasis and cholecystitis (see Q19). However, the conservative approach appears to be successful in a minority of cases (32 respondents (63%) answered that in 0–20% of cases a conservatory treatment was successful (see Q18)). In case of an acute cholecystitis, 22% of participants conduct a cholecystectomy within the first 24 h of symptoms (n = 11). Further two participants (4%) proposed a cholecystectomy within 48 h of symptoms in the free text. 57% of participants preferred an elective approach of surgery, irrespective of presence of a complicated cholelithiasis (n = 29). In the open question Q20 we asked for temporal limits for the surgical indication and individual standardized approaches. An explicit case-by-case-decision without a standardized approach was reported by 6 participants (12%). Further 6 participants (12%) specified a time slot of 6 months or no temporal limitation. Two participants (4%) recommended adherence to the adult guideline.

### Diagnostic and therapeutic algorithm

**Fig. 3:**
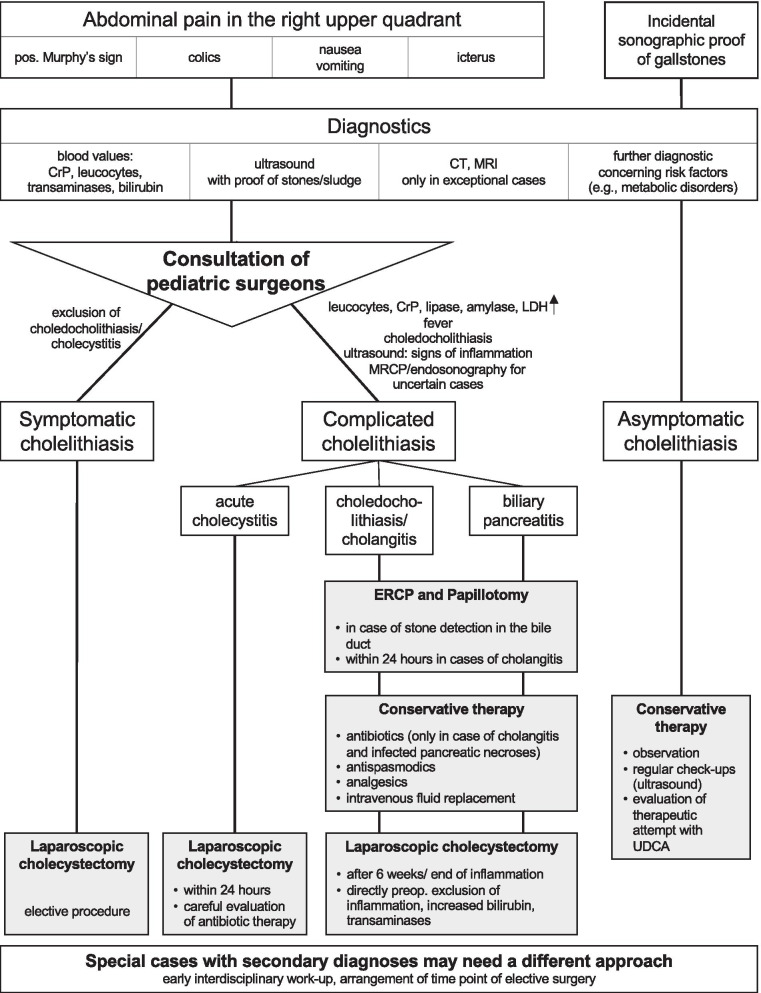
Diagnostics and Therapy Flow Chart. A draft of a flow chart based on close teamwork between pediatricians and pediatric surgeons for optimal diagnostic and therapeutic algorithm until evidence-based pediatric guidelines are available in pediatric cholelithiasis. A primarily pediatric medical admission is assumed

The assessment of the retrospective study’s and the survey’s findings prompted the development of a diagnostic and therapeutic proposal in accordance with the adult guidelines due to the lack of large-scale multi-center studies in pediatric patients. This algorithm is depicted in Fig. 3. It emphasizes the close and early interdisciplinary consensus of pediatricians and pediatric surgeons, as well as the clear differentiation between symptomatic and asymptomatic cholelithiasis and complicated cholelithiasis in cases of acute inflammation or choledocholithiasis. We propose to discuss cases with concomitant diseases early and interdisciplinary due to the continued lack of large-scale population studies for evidence-based guidelines. It certainly cannot provide answers to all therapeutic options (e.g., antibiotic therapy), as large-scale population studies are missing, and information has to be transferred from adult management.

## Discussion

Whereas cholelithiasis is one of the most widespread diseases in adult visceral surgery, only little is known about specific factors of cholelithiasis and its complications in children and adolescents. We therefore primarily conducted a retrospective analysis of our own patients’ cohort, differentiating between pediatric and adult management. While an elevated BMI is estimated as one of the important risk factors in the development of adult cholelithiasis [16], other diagnoses such as metabolic disorders, parenteral nutrition, cystic fibrosis, hemolytic diseases and malignancies are strongly correlated with pediatric gallstones [17–19]. We can confirm this through the findings of the retrospective analysis, as concomitant diseases are correlated with the pediatric surgery subgroup and obesity with the visceral surgery subgroup. Etiology and predisposing factors of cholelithiasis in pediatric patients are recommended to be discussed carefully [2]. They may ultimately have an influence on interdisciplinary therapeutic decisions and management should be discussed within a pediatric guideline.

We are presenting this study with the specific aim of exploring especially the therapeutic management and the surgical timing in pediatric cases of cholelithiasis. Findings of the retrospective study indicate a preferred expectant management in pediatric patients with an increased time span with symptoms and longer timeframe between diagnosis and surgery. This may be largely determined by the high proportion of primary pediatric medical admissions. Overall percentage of complicated cases was not significantly higher in pediatric patients in comparison to patients of the visceral surgery subgroup. However, we see an increased risk for complications, especially for biliary pancreatitis, in these patients besides the unnecessarily extended duration of symptoms in pediatric patients. The preference for an expectant management in symptomatic or even acute and complicated cases was also reported within the online survey. Based on the results of the dual approach of our study, we can conclude that the management practice is heterogeneous and deviates fundamentally from the guidelines of adult medicine [8]. Moreover, the survey’s findings revealed a lack of standardization or homogeneous optimal surgical timing after end of conservative treatment. The ideal surgical timing has been widely discussed in adult medicine so far, which could be partially solved by the guideline for adults [8]. Various publications appear to be in consensus on the association of delayed treatment with increased complication rates. However, certain limitations, such as restricted case numbers, the high risk of selection bias and inconsistent definitions of diagnoses, must be taken into account to properly classify the value of these publications. Sarrami et al. demonstrated in a retrospective analysis of 188 children and adolescents with cholelithiasis that the risk of subsequent hospital admission was increased by 5% for every 10 days with delayed treatment [3]. They speculated that a delay in surgery might increase complication rates, while acknowledging their limitations of an inconsistent definition and therapeutic approach in patients with symptomatic cholelithiasis and the need for large, randomized trails.

Additionally, Curro et al. proposed to conduct cholecystectomies even in cases of asymptomatic cholelithiasis in patients with sickle cell disease. In symptomatic patients, association with increased operative time, morbidity rate, and postoperative stay could be revealed [20]. However, these results are based on an observational study within a restricted number of 30 patients with cholelithiasis and sickle cell disease. Tannuri et al. observed within their retrospective study with the large time period of 17 years a high percentage of 25% of patients, presenting directly with a complication due to cholelithiasis [9]. Pelizzo et al. most recently identified new diagnostic scores in a small population study to improve surgical timing [4]. They see however that the lack of certain clinical background data causes a selection bias and therefore limits the interpretation of results. We observed 55% of the pediatric patients within our retrospective study being operated on in a time period of day 5–42 after acute signs of inflammation. In the pediatric subgroup, only one patient with acute cholecystitis was treated with a cholecystectomy within 24 h. The inflammatory process within this time slot might unnecessarily complicate surgeries, elongate operation time and cause intra- and postoperative complications. Recommendations of the adult guideline are thus not implemented and their respective value in pediatric management should be carefully discussed.

Furthermore, with this given preference for expectant management, the value of UDCA in pediatrics has to be carefully evaluated. According to the S3-guidelines [8], therapy with UDCA is recommended in asymptomatic patients. However, in pediatric practice, a relevant number of patients with symptomatic cholelithiasis receives UDCA for litholysis. Our results confirm the broad range of application in pediatric management without sufficient success. In 2008, Della Corte et al. claimed the ineffectiveness of UDCA in treatment of cholelithiasis, even though a significant relief of symptoms could be reported [21]. This reduction of symptoms is also confirmed by Baran et al., who reported a study of 74 children, mostly responding to UDCA treatment within the first six months [22]. Larger population studies are required to evaluate the real value of UDCA in pediatric patients.

The establishment of evidence-based pediatric guidelines is required in order to reduce insecurities and to deliver scientific facts on the basis of multicenter and prospective studies. These should not only focus on a diagnostic algorithm, but especially on the indications for expectant management versus surgical therapy and further therapeutic options in acute and chronic disease. In an effort to achieve an improved standardized treatment and to emphasize the consensus between pediatricians and pediatric surgeons until results of evidence-based pediatric guidelines are available, we illustrated a flow chart based on our findings and to encourage common strategies (Fig. 3). We especially excluded different marginal subgroups of patients, e.g., symptomatic patients with no clear association between symptoms and cholelithiasis. These may be the subject of subsequent research projects.

Limitations of the study have to be discussed carefully. Aimed at gaining insight into real therapeutic management, we are presenting a foundation for further research and are not able to add results of a large prospective study on this topic. Accordingly, one limitation of our presented retrospective research lies in the relatively small study population over a large period of time, which limits the informative value of our study. However, it reflects the still low incidence. Although representing the diverse patient spectrum in patients with cholelithiasis, the case mix and the variety of complications additionally limit the possibility to draw strong conclusions because of even smaller case numbers within subgroup analyses. Nevertheless, statistically significant differences between the two study groups have been obtained for several parameters of clinical relevance. Certainly, a type II error cannot be excluded for non-significant test results because of the minor statistical power. Furthermore, challenges in the interpretation of data are caused by two aspects of the study design. First, analysis of complicated cases is of special interest and must be seen with caution, as they are analyzed separately and within the whole study group. We aimed at distinguishing between these subgroups to emphasize the need for different management and surgical timing. Second, the study’s approach was chosen from the therapeutic endpoint of cholecystectomy, which influences the selection of patients (bias). This study design was chosen based on rising numbers of cholecystectomies, which were not attributable to a changed therapeutic regimen with earlier indication for surgery. Finally, our study does therefore not include any aspects on the effects of an expectant management in asymptomatic cases, which might certainly be a relevant subgroup of patients with cholelithiasis. The presented online survey additionally showed a restricted response rate, but is in the range of other published online surveys [23]. It was designed not to report exact numbers, but to deliver an insight in the current management and to create awareness for the lack of a guideline. This first ever online survey on this topic might therefore hold a high risk of recollection bias, as we asked for individual characteristics in practices. However, even from this restricted point of view, we observed a heterogeneous picture in the pediatric management with a tendency of delayed timing of surgery. We propose to conduct further prospective multicenter studies and international consensus conferences to further explore single aspects on diagnostics and therapy.

## Conclusion

In conclusion, the actual treatment of cholelithiasis and cholecystitis in children and adolescents is characterized by a diagnostic and therapeutic heterogeneity. Future aims should focus on an early interdisciplinary cooperation and a work consensus between pediatricians and pediatric surgeons. With this publication, we would like to present an insight into real therapeutic management and to emphasize the need for large population-based, prospective studies. The development of evidence-based pediatric guidelines may represent a key factor in treating this increasing entity sufficiently and in an improved and homogeneous way.

## Supplementary information

**Additional file 1**. Survey on Treatment of symptomatic cholecystolithiasis / cholecystitis in patients under 18 years of age

## Data Availability

The datasets used and/or analyzed during the current study are available from the corresponding author on reasonable request.

## Abbreviations

BMI: body mass index
CL: cholelithiasis
ERCP: endoscopic retrograde cholangiopancreatography
MRCP: magnetic resonance cholangiopancreatography
MRI: magnetic resonance imaging
PCL: pediatric cholecystolithiasis
UDCA: ursodesoxycholic acid
