# Supplementary material for: Cholelithiasis and cholecystitis in children and adolescents: Does this increasing diagnosis require a common guideline for pediatricians and pediatric surgeons?
Source: BMC Gastroenterol. 2021 Apr 21;21:186. doi: 10.1186/s12876-021-01772-y (PMC8061037; doi:10.1186/s12876-021-01772-y)
Supplement: Supplementary file 1 — Additional file 1. Survey on Treatment of symptomatic cholecystolithiasis / cholecystitis in patients under 18 years of age [file 12876_2021_1772_MOESM1_ESM.pdf]

Survey on Treatment of symptomatic cholecystolithiasis / cholecystitis in patients under 18 years of age

1. Which city is your clinic/MVZ/practice located in?
   Free text

2. Do you work at a
   - University hospital
   - (Non-university) hospital
   - MVZ/practice with staff beds
   - Other: *free text*

The following questions regarding demographics refer to the treatment period of the last 5 years:

3. How many cases of symptomatic cholecystitis under the age of 18 have you treated in the last 12 months?
   - None
   - Up to 5 cases
   - 5-10 cases
   - 10-20 cases
   - 20-30 cases
   - 30 cases

4. Is the clinical picture of cholecystitis / symptomatic cholecystolithiasis primarily treated by pediatricians and then presented surgically, or is referral made directly to your pediatric surgeons?
   - Predominantly primary pediatric presentation
   - Predominantly primary surgical presentation

5. What led to the diagnosis?
   - The majority of diagnoses is based on typical clinical findings
   - The majority of diagnoses is based on imaging/laboratory incidental findings

6. What are the age groups of your patients with symptomatic cholecystolithiasis/ cholecystitis? *Multiple answers possible*.
   - Newborns
   - Infants (until end of 1st year of life)
   - 1-6 years
   - 6-8 years
   - 8-10 years

- 10-13 years
- 13-18 years

7. Which gender predominates among patients with symptomatic cholecystolithiasis / cholecystitis?
    - Predominantly female patients
    - Predominantly male patients

8. What is the proportion of your patients with symptomatic cholecystolithiasis / cholecystitis from the Mediterranean region?
    - 0 – 20 %
    - 20 – 40 %
    - 40 – 60 %
    - 60 – 80 %
    - 80 – 100 %

The following questions regarding etiology and diagnosis refer to the treatment period of the last 5 years:

9. What are the concomitant disorders of affected children with symptomatic cholecystolithiasis / cholecystitis? *Multiple answers possible*.
    - Obesity
    - CF
    - Celiac disease
    - Other metabolic diseases (free text with common diseases, if applicable)
    - Malignant diseases/chemotherapy
    - Organ transplantation/immunosuppression
    - Hemolytic diseases (anemia, spherocystosis)
    - Parenteral nutrition
    - Liver failure
    - Other diseases: *free text*

10. Is there a noticeable increase in the percentage of children and adolescents with obesity and gallstone disease in your clinic?
    - Yes
    - No

11. Do you consider clarification of possible causative diseases/metabolic disorders in symptomatic cholecystolithiasis / cholecystitis important?
    - Yes
    - No

12. Does cholecystitis occur simultaneously at the time of diagnosis of symptomatic cholecystolithiasis?
    - Predominantly yes
    - Predominantly no

13. Are the clinical signs of jaundice present at initial diagnosis of your patients with symptomatic cholecystolithiasis / cholecystitis?
    - Predominantly yes
    - Predominantly no

14. Is a biliary pancreatitis present at initial diagnosis?
    - Predominantly yes
    - Predominantly no

15. Has a gallstone ileus ever been observed in your patients at your center?
    - Yes
    - No

16. What imaging diagnostics do you routinely perform to diagnose symptomatic cholecystolithiasis / cholecystitis? *Multiple answers possible*.
    - Sonography
    - CT
    - MRI
    - Other

17. Is an endoscopic retrograde cholangiopancreatography (ERCP) performed as part of diagnostics and therapy?
    - Yes, always
    - Predominantly yes
    - Rarely
    - Never

    Indications for ERCP in your center? *Free text*

The following questions regarding therapy refer to the treatment period of the last 5 years:

18. What percentage with symptomatic cholecystitis was successfully treated conservatively?
    - 0 – 20 %
    - 20 – 40 %
    - 40 – 60 %
    - 60 – 80 %
    - 80 – 100 %

19. What is the standard conservative treatment for symptomatic cholecystolithiasis / cholecystitis in children in your hospital? *Multiple answers possible*.
    - o Antibiotics
    - o Spasmolytics
    - o Food restriction
    - o Analgesics
    - o Ursodeoxycholic acid (UDCA)

    Does your clinic/hospital deploy other elements of standard conservative treatment?
    Free text

20. What do you consider to be the time limit for the continuation of conservative therapy?
    Free text

21. What was your procedure in the acute phase of the disease?
    - o Surgery within 24 hours after presentation at the clinic
    - o Surgery within 6 weeks
    - o Are there any other standards in your surgical procedure? *Free text*

22. Which discipline operates on patients with cholecystitis / symptomatic cholecystolithiasis in your hospital?
    - o Pediatric surgery
    - o General surgery
    - o General surgery is only involved in complicated cases.

23. What is the procedure for cholecystectomies in your hospital? *Multiple answers possible*.
    - o Always laparoscopic approach
    - o Predominantly laparoscopic approach
    - o Balanced ratio open access - laparoscopy
    - o Predominantly open access
    - o Always open access
    - o Conversion is necessary in a high percentage

24. Is an analysis of the stones performed as part of the histopathological examination?
    - o Yes
    - o No
